# Post-stroke Depression, Functional Independence, and Cognition Among Patients With High Social Support in Georgia: A Cross-Sectional Study

**DOI:** 10.7759/cureus.89790

**Published:** 2025-08-11

**Authors:** Aaryan M Kapoor, Aman Ansari, Nina Otinashvili, Dhanin Shanly, Nikoloz Jikia, Janet George, Tamuna Akhvlediani

**Affiliations:** 1 Faculty of Medicine, Tbilisi State Medical University, Tbilisi, GEO; 2 Department of Neurology, Georgian American University, Tbilisi, GEO

**Keywords:** cognitive impairment, functional disability, post-stroke depression, rehabilitation, social support

## Abstract

Objectives: The study aims to assess the relationships between depressive symptoms and functional as well as cognitive outcomes in Georgian stroke patients who report a high perception of social support (SS).

Methods: A cross-sectional analysis was conducted between September and December 2024 at the Ken Walker Hospital and Aversi Rehabilitation Center. A total of 33 post-stroke patients with a high perception of social support as measured by the Multidimensional Scale of Perceived Social Support (MSPSS) were selected and assessed for depression using the Patient Health Questionnaire-9 (PHQ-9). Cognitive function and functional independence scores were obtained from the respective institutions' medical databases. Spearman's correlation and multivariate linear regression were used for data analysis.

Results: Functional independence showed a strong inverse correlation with depressive symptoms (r = -0.69, p < 0.001), and cognitive performance correlated moderately and inversely with depression severity (r = -0.52, p = 0.008) in patients with a high perception of social support. Regression analysis, when controlled for age and sex, showed that higher PHQ-9 scores were significantly associated with lower Barthel Index (β = -0.1987, p =0.001) and MoCA scores (β = -1.0589, p = 0.004), supporting findings from bivariate analyses.

Conclusions: High perception of social support did not appear to "buffer" the association between post-stroke depression (PSD) and poor functional or cognitive outcomes, particularly within the sociocultural context of the country of Georgia. This cultural context of strong familial support and traditional societal roles is particularly understudied and is similar to those seen in several other Eastern European/Asian countries. Larger longitudinal studies are needed to clarify causal pathways and eliminate all possible confounders.

## Introduction

A cerebrovascular stroke is caused by an acute loss of blood flow to the brain, resulting in a sudden onset of neurologic deficits. Stroke survivors are often referred for neuropsychiatric evaluation during all phases of recovery to manage different psychiatric sequelae of the disease [1]. Post-stroke depression (PSD) is one such complication that worsens functional disability (FD), increases the risk of mortality, and delays recovery [2].

A study found a significant correlation between depressive symptoms post-stroke and FD, wherein 20% of patients with moderate depressive symptoms had severe disability, compared to 40% of those with severe depressive symptoms [3]. Stroke survivors with depression have also been shown to have lower functional independence in activities of daily living (ADLs) when compared with non-depressed individuals. Depressive symptoms, such as lack of motivation, fatigue, and impaired cognition, all contribute directly or indirectly to greater disability and a diminished quality of life [4].

PSD is also associated with a higher risk of cognitive impairment (CI) when compared to non-depressed individuals, and this further reiterates the importance of a neuropsychiatric referral in stroke patients [5]. These deficits are especially pronounced in major areas of memory and executive functioning [6]. Psychosocial factors, such as reduced social support (SS) and decreased involvement in cognitive activities, exacerbate cognitive decline. Furthermore, the presence of PSD decreases the chances of participating in rehabilitation programs, hence limiting cognitive recovery [7].

The relationship between SS and PSD seems intuitive, with a high SS being associated with decreased PSD; however, studies have reported conflicting results. Some research has indeed found a significant inverse correlation, whereas others found that although SS was not significantly associated with PSD, it played a role in mediating the relationship between the ability to perform ADLs and PSD [8,9].

To better study the mediating role of SS in those relationships, our study focuses on the unique subgroup of post-stroke patients who report high levels of perceived SS. We aim to examine whether depression in this group remains associated with the poor ability to independently perform ADLs as well as poor cognitive ability. Our theoretical focus is on whether the detrimental association of PSD on outcomes is offset by the protective effects of high SS.

Further, our study is a novel take on examining this relationship in the context of Georgia, a country with a strong emphasis on social relationships and a prominent culture of interdependency and community support; this context is commonly seen in many Eastern European/Asian countries with similar socioeconomic status. This population remains underrepresented in medical academia due to the financial limitations, systemic issues, and operational obstacles, thus highlighting the relevance and potential contributions of our study [10].

## Materials and methods

Cross-sectional analysis was conducted between September and December 2024 at Ken Walker Hospital and Aversi Rehabilitation Center. Data was collected from patients assigned to sub-acute rehabilitation after stroke. Montreal Cognitive Assessment (MoCA) and Barthel Index were administered in the Georgian language by neurologists at the respective institutions on the first day of sub-acute rehabilitation, which occurred between 14 and 90 days following stroke diagnosis as part of the routine intake procedure. Multidimensional Scale of Perceived Social Support (MSPSS) and Patient Health Questionnaire-9 (PHQ-9) data were also collected within this timeframe. A minimum period of 14 days was selected based on the specific institutional regulations, which stipulated that patients become eligible for admission to sub-acute rehabilitation programs only after 14 days have passed since the initial stroke diagnosis.

Inclusion criteria involve patients diagnosed with cerebrovascular stroke and MSPSS score greater than 61, indicating a high perception of SS [11]. Patients must be able to understand and communicate in the local language or have access to an interpreter. Patients must be ≥18 years old. Exclusion criteria include patients with preexisting depression or other psychiatric disorders, severe CI, MSPSS scores < 61, and significant aphasia or inability to communicate, and patients who do not give informed consent.

Ethics board approval was obtained from the Research Ethics Committee of Tbilisi State Medical University (TSMU) with ethics code #5-2024/112-4.9, and permission was granted from the respective centers.

A total of 41 patients were interviewed; five were excluded due to MSPSS < 61, two due to significant aphasia, and one due to severe CI. The remaining 33 patients participated in the study (N = 33) and met our inclusion criteria.

The MSPSS is a questionnaire that measures an individual's perception of SS from three sources: family, friends, and a significant other. It measures the individual's perception of the SS available to them, specifically emotional support, perceived availability, and appraisal support. It does not measure material support, such as financial or institutional support. The tool assesses the availability and quality of SS through a 12-item questionnaire rated on a 7-point Likert scale, with a lower score indicating decreased perceived SS [11].

The PHQ-9 is a widely used metric for screening and diagnosing depression in various settings. It consists of nine questions that align with the Diagnostic and Statistical Manual of Mental Disorders, 5th Edition criteria for major depressive disorder (MDD). Total scores range from 0 to 27, with each item being scored from 0 to 3 in terms of frequency of symptoms, with 0 being "not at all" to 3 being "nearly every day." The results stratify the severity of depression into five levels: minimal or no depression (0-4), mild (5-9), moderate (10-14), moderately severe (15-19), and severe (20-27). The tool is quick to administer and provides a clear, quantifiable measure of depression severity [12].

The Barthel Index is a scale used for quantifying functional independence in ADLs, commonly in the context of rehabilitation for stroke patients. It assesses various activities, such as feeding, bathing, dressing, mobility, and bowel and bladder control, and scores range from 0 (complete dependence) to 100 (full independence). This scale can be used to measure functional impairment, plan rehabilitation goals, and track recovery progress [13].

The MoCA is a screening tool for assessing cognitive function and is used for detecting cognitive decline/impairment in neurologic conditions such as dementia and stroke. Cognitive domains such as attention, executive function, language, memory, abstract thinking, visuospatial skills, and orientation are evaluated by the tool. It is scored out of 30, with a score above 26 being considered negative for CI [14].

Using Graphpad Prism version 10.5.0 (Dotmatics, Boston, MA), a Spearman's correlation was used to find the association between PHQ-9 and Barthel Index, as well as PHQ-9 and MoCA, in patients who met the inclusion criteria of MSPSS > 61. A multivariate linear regression was also performed between those indices to account for age and sex as confounders. Analysis was performed using Python version 3.13.5 (Python Software Foundation, Wilmington, DE), primarily employing the statsmodels and scikit-learn libraries.

## Results

Our sample consisted of 33 post-stroke patients currently undergoing rehabilitation, with five patients from Aversi Rehabilitation Center and 28 patients from the Ken Walker Hospital (Table 1). Of the 33 patients, 24 (72%) presented with depressive symptoms ranging from mild to severe, and nine (28%) presented with minimal to no depressive symptoms, with mean PHQ-9 scores of 9.03 ± 5.63 (Table 2).

**Table 1:** Participant demographics SD: standard deviation

Variable	Total (N = 33)	Male (n = 25)	Female (n = 8)
Age (mean ± SD)	62.6 ± 10.2	62.6 ± 9.5	62.6 ± 12.8
Age median	62	62	62.5
Age mode	70	70	40
Age range	40-80	45-78	40-80

**Table 2:** Descriptive statistics: PHQ-9, Barthel Index, and MoCA scores PHQ-9: Patient Health Questionnaire-9, MoCA: Montreal Cognitive Assessment, SD: standard deviation

Variable	PHQ-9	Barthel Index	MoCA
Distribution	Non-normal	Normal	Non-normal
Mean ± SD	9.03 ± 5.63	76 ± 17.05	24.61 ± 2.75
25th percentile	4	70	23
Median	9	78	26
75th percentile	14.5	87.5	27
Interquartile range	10.5	17.5	4

**Figure 1:**
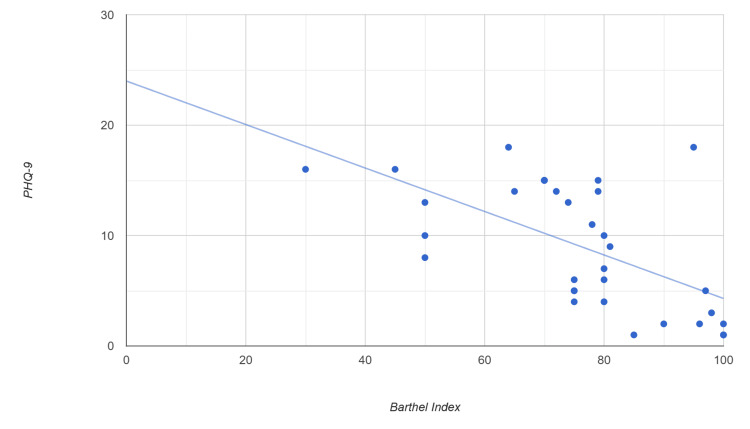
Scatterplot of Barthel Index versus PHQ-9 score Each point represents one individual. The Spearman correlation coefficient is r = -0.56 (p = 0.001). A linear regression line is plotted with the equation: PHQ-9 = -0.197 × Barthel Index + 23.995, indicating a moderate negative trend. PHQ-9: Patient Health Questionnaire-9

A Spearman's correlation analysis between Barthel Index and PHQ-9 scores revealed a moderate to strong negative association (r = -0.688, p < 0.001), indicating a statistically significant inverse relationship (Figure 1). To explore further, as well as control for potential confounders, a multivariate linear regression was conducted with PHQ-9 as the dependent variable and the Barthel Index as a predictor, adjusting for age and sex. The analysis yielded a regression coefficient (β) of -0.1987 (p = 0.001), supporting the bivariate analysis (Table 3).

**Table 3:** Multivariate linear regression between Barthel Index and PHQ-9 scores, controlling for age and sex PHQ-9: Patient Health Questionnaire-9, CI: confidence interval

	Regression coefficient (β)	Standard error	t	p-value	95% CI lower	95% CI higher
Constant	26.5789	7.028	3.782	0.001	12.205	40.953
Barthel Index	-0.1987	0.052	-3.847	0.001	-0.304	-0.093
Age	-0.0397	0.083	-0.481	0.634	-0.209	0.129
Sex	0.1427	2.009	0.071	0.944	-3.966	4.251

**Figure 2:**
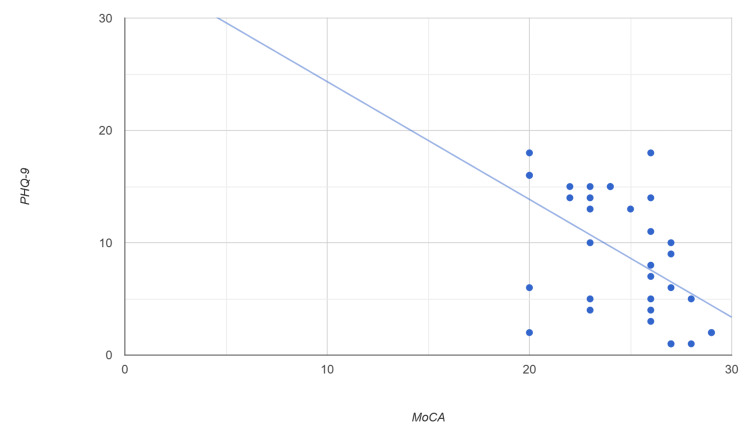
Scatterplot of MoCA score versus PHQ-9 score Each point represents an individual participant. The Spearman correlation coefficient was r = -0.47 (p = 0.006). A linear regression line is shown with the equation: PHQ-9 = -1.048 × MoCA + 34.829, indicating a moderately negative linear trend. PHQ-9: Patient Health Questionnaire-9, MoCA: Montreal Cognitive Assessment

Likewise, Spearman's correlation between MoCa and PHQ-9 scores across all participants showed a moderately negative relationship (r = -0.522, p = 0.008) (Figure 2). This highly significant result indicates that lower cognitive scores are associated with higher levels of depressive symptoms. Similarly, regression analysis was repeated with PHQ-9 and MoCA, controlling for age and sex, yielding a β of -1.0589 (p = 0.004), further confirming our findings (Table 4).

**Table 4:** Multivariate linear regression between MoCA and PHQ-9 scores, controlling for age and sex PHQ-9: Patient Health Questionnaire-9, MoCA: Montreal Cognitive Assessment, CI: confidence interval

	Regression coefficient (β)	Standard error	t	p-value	95% CI lower	95% CI higher
Constant	38.4821	10.982	3.504	0.002	16.021	60.944
MoCa	-1.0589	0.335	-3.165	0.004	-1.743	-0.375
Age	-0.0591	0.089	-0.666	0.511	-0.241	0.122
Sex	1.243	2.067	0.601	0.553	-2.986	5.47

## Discussion

Our findings show that moderate to strong inverse correlations exist between PSD and both FD and CI, respectively, in patients who report a high perception of SS. These significant findings suggest that despite high perceived non-material support, the associations between PSD and impaired cognitive and functional outcomes remain substantial. This study hypothesizes that the buffering effects of SS may not be protective enough to alleviate the impact of PSD on functional and cognitive status or vice versa.

To elucidate the relationship between social support and FD, a review across the existing literature suggests that SS, including non-material and emotional support, affects FD in more ways than one. Strengthening support from family members and health personnel may be one of the decisive factors in improving the quality of life of stroke patients [15]. Interestingly, some studies say that peer support, something we did not distinguish between, may even be more helpful than familial support when coping with physical limitations [16].

Despite the aforementioned studies highlighting the significance of SS, it cannot fully compensate for physical impairments, as they significantly affect an individual's participation in social life and their independence, as highlighted by a study done in 2022 [17]. Reduced social participation has been linked to depression bidirectionally, both as a cause and a consequence [18]. This may help explain why, in our sample, participants with high perceived social support still demonstrated significantly reduced functional independence in the context of post-stroke depression.

The idea that high social support correlates with reduced CI is described in the literature, but it is not as straightforward as one might expect. One proposed mechanism is that social interaction requires enhanced cognitive activity, which in turn enhances neural connectivity and fosters brain plasticity, improving overall cognitive function [19]. Furthermore, just the presence or absence of social support is not enough to determine cognitive outcomes. A study done in 2020 found that individuals who perceived their SS as insufficient were significantly more likely to report cognitive decline, highlighting the importance of perception when it comes to social support [20]. This perception can be further affected in patients suffering from depression who could negatively view existing support due to their psychopathology.

Interestingly, some researchers found no significant differences in CI between those with or without identifiable support, similar to our findings, wherein CI remained closely associated in PSD states despite a high perception of support. However, a caveat to this is that the aforementioned study focused on identifiable support being more aligned with physical support rather than emotional, an aspect our study did not explore. This further advances the idea that not all types of support are considered equal, and one may play a more significant role than the other [17].

CI remains an important aspect in the care of post-stroke patients, especially with there being a relationship between PSD and a risk of dementia in the long term. For example, a Polish study suggests individuals with depressive symptoms after a stroke had a greater probability of developing dementia within five years in comparison to those without depression [21]. This reinforces the essential role of early intervention in PSD patients to help mitigate cognitive decline, along with enhancing immediate improvements in mental health.

FD after a stroke is a key indicator of both psychological distress and cognitive decline, with studies demonstrating that individuals with marked FD post-stroke are at an independently increased risk for developing dementia [22]. Furthermore, this correlation is bidirectional, with FD not being the only contributor to the onset of dementia; CI can also compromise recovery from FD, creating a vicious cycle that exacerbates both conditions [23]. This cyclical relationship underscores the necessity for integrated rehabilitation strategies that address both physical and cognitive aspects of recovery.

While we have discussed the associations of PSD with FD and CI, the nature of their relationship, including longitudinal effects and causality, remains unclear [24,25].

Cultural factors, including the role of social support, have also been shown to mediate the relationship between PSD and functional outcomes. Emerging intervention strategies must therefore account for cultural constructs of disability and help-seeking behaviors, particularly in transitional societies like Georgia, where traditional family support networks intersect with evolving rehabilitation paradigms [26,27]. By addressing these gaps through robust longitudinal and culturally sensitive research, the field can move toward more personalized and effective rehabilitation strategies for stroke survivors.

Our study's strengths include its focus on an understudied Georgian population representing a lower-middle-income community characterized by strong social responsibility, communal values, and notable mental health stigma. A culture of traditional family roles, often with a single income earner and widespread fear of social rejection due to mental health concerns or disability, makes the intersection of PSD and SS particularly compelling to study in this context. Further, our study is a novel approach to isolating the effects of SS and the detrimental effects of PSD on functional and cognitive ability, which is an understudied aspect of stroke management and rehabilitation, opening avenues for different elements of post-stroke rehabilitation.

Our study involves several limitations. The cross-sectional design of our study precludes causal interference between PSD and FD/CI, making directionality out of the scope of our research. The lack of a comparison group with low SS in our study design limits our ability to measure precise protective measures of SS in the relationship of PSD and FD/CI. While we performed a multivariate regression analysis to control for age and sex as confounders, we were unable to account for other potential confounders, such as stroke severity, lesion location, and length of hospital stay, due to varying standards of care in the country and resource roadblocks that prevent our centers from adequately collecting that data. We have also not distinguished between different sources of support, such as family, peers, or religious institutions. Lastly, the smaller sample size may have contributed to the potential skewing of our findings.

## Conclusions

Our study presents a negative correlation that exists between PSD and FD/CI in patients with a high perception of SS. Our study is a novel take on the relationship of these factors in this specific patient population. We found that a high perception of SS was unable to act as a "buffer" for FD and CI in patients with PSD, leading us to question its effects on a patient's functional and cognitive health. We bring further nuance by studying this association in an understudied Georgian population with social dynamics that are similarly found across Eastern Europe/Asia.

Future research should adopt longitudinal designs to demonstrate the causal links between functional recovery, cognitive function, and post-stroke depression (PSD) over time. Furthermore, larger, multicenter studies could define more precisely subgroup-specific effects, such as gender or cultural influences, which have shown inconsistent results in smaller cohorts like ours.

## Figures and Tables

**Figure 3:**
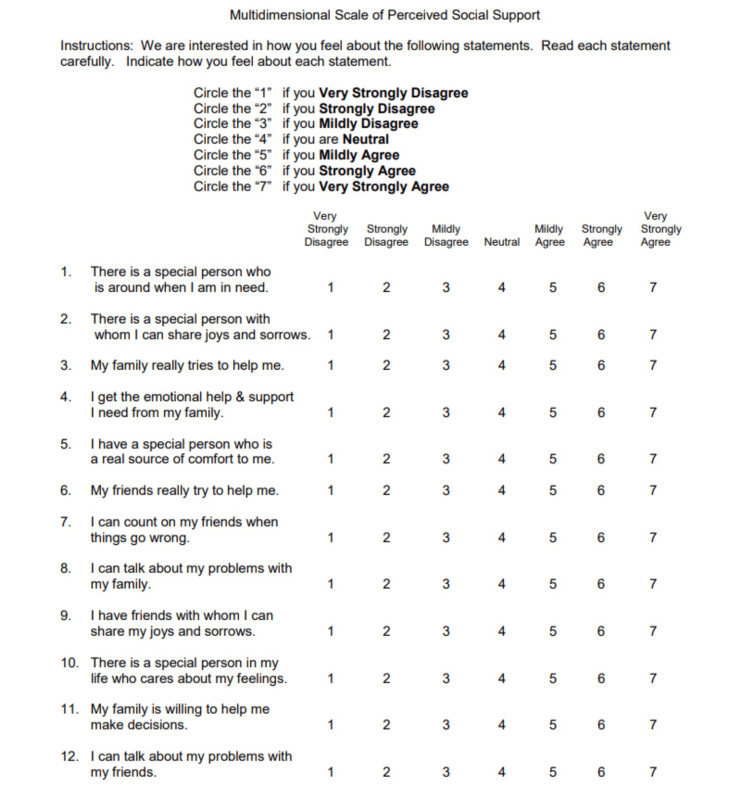
MSPSS MSPSS: Multidimensional Scale of Perceived Social Support Zimet GD, Dahlem NW, Zimet SG, Farley GK: The Multidimensional Scale of Perceived Social Support. J Pers Assess. 1988, 52:30-41. 10.1207/s15327752jpa5201_2 [11] Open access, no permission required to reproduce, translate, display, or distribute

**Figure 4:**
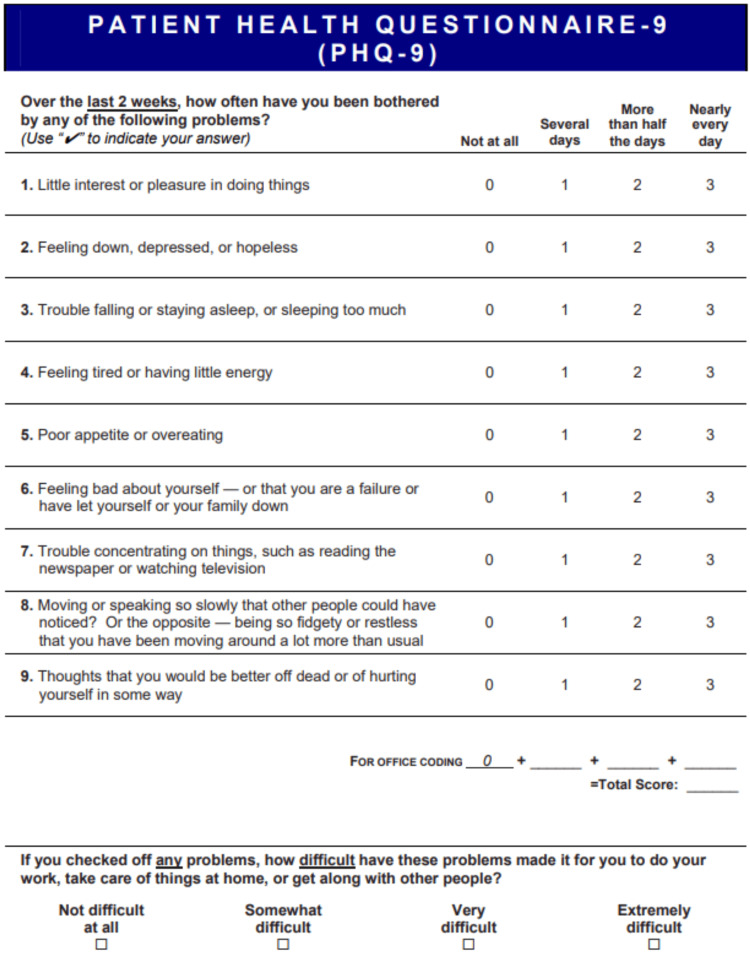
PHQ-9 PHQ-9: Patient Health Questionnaire-9 Kroenke K, Spitzer RL, Williams JB: The PHQ-9: validity of a brief depression severity measure. J Gen Intern Med. 2001, 16:606-13. 10.1046/j.1525-1497.2001.016009606.x [12] Open access, no permission required to reproduce, translate, display, or distribute

## Appendices

Figure 3 and Figure 4 present the Multidimensional Scale of Perceived Social Support questionnaire and Patient Health Questionnaire-9, respectively.

## References

[1] (2023). Neuropsychological Interviewing of Adults. Neuropsychological Interviewing of Adults.

[2] Chemerinski E, Robinson RG (2000). The neuropsychiatry of stroke. Psychosomatics.

[3] Astuti P, Kusnanto K, Dwi Novitasari F (2020). Depression and functional disability in stroke patients. J Public Health Res.

[4] Ezema CI, Akusoba PC, Nweke MC, Uchewoke CU, Agono J, Usoro G (2019). Influence of post-stroke depression on functional independence in activities of daily living. Ethiop J Health Sci.

[5] Kauhanen M, Korpelainen JT, Hiltunen P (1999). Poststroke depression correlates with cognitive impairment and neurological deficits. Stroke.

[6] Kusec A, Demeyere N (2025). Relationship of subjective and objective cognition with post-stroke mood differs between early and long-term stroke. Clin Neuropsychol.

[7] Elayoubi J, Haley WE, Nelson ME, Hueluer G (2023). How social connection and engagement relate to functional limitations and depressive symptoms outcomes after stroke. Stroke.

[8] Babkair LA, Chyun D, Dickson VV, Almekhlafi MA (2021). The effect of psychosocial factors and functional independence on poststroke depressive symptoms: a cross-sectional study. J Nurs Res.

[9] Huang CY, Hsu MC, Hsu SP, Cheng PC, Lin SF, Chuang CH (2010). Mediating roles of social support on poststroke depression and quality of life in patients with ischemic stroke. J Clin Nurs.

[10] Woods WA, Watson M, Ranaweera S, Tajuria G, Sumathipala A (2023). Under-representation of low and middle income countries (LMIC) in the research literature: ethical issues arising from a survey of five leading medical journals: have the trends changed?. Glob Public Health.

[11] Zimet GD, Dahlem NW, Zimet SG, Farley GK (1988). The Multidimensional Scale of Perceived Social Support. J Pers Assess.

[12] Kroenke K, Spitzer RL, Williams JB (2001). The PHQ-9: validity of a brief depression severity measure. J Gen Intern Med.

[13] Collin C, Wade DT, Davies S, Horne V (1988). The Barthel ADL Index: a reliability study. Int Disabil Stud.

[14] Nasreddine ZS, Phillips NA, Bédirian V (2005). The Montreal Cognitive Assessment, MoCA: a brief screening tool for mild cognitive impairment. J Am Geriatr Soc.

[15] Butsing N, Tipayamongkholgul M, Ratanakorn D, Suwannapong N, Bundhamcharoen K (2019). Social support, functional outcome and quality of life among stroke survivors in an urban area. J Pac Rim Psychol.

[16] Kwok SY, Yeung DY, Chung A (2011). The moderating role of perceived social support on the relationship between physical functional impairment and depressive symptoms among Chinese nursing home elderly in Hong Kong. ScientificWorldJournal.

[17] Ashaie SA, Castro N (2022). Complexity of participation post-stroke: longitudinal assessment of community participation, positive affect, social support and functional independence. J Rehabil Med.

[18] Silva SM, Corrêa JC, Pereira GS, Corrêa FI (2019). Social participation following a stroke: an assessment in accordance with the international classification of functioning, disability and health. Disabil Rehabil.

[19] Ma T, Liao J, Ye Y, Li J (2024). Social support and cognitive activity and their associations with incident cognitive impairment in cognitively normal older adults. BMC Geriatr.

[20] Weng X, George DR, Jiang B, Wang L (2020). Association between subjective cognitive decline and social and emotional support in US adults. Am J Alzheimers Dis Other Demen.

[21] Filipska K, Wiśniewski A, Biercewicz M, Ślusarz R (2020). Are depression and dementia a common problem for stroke older adults? A review of chosen epidemiological studies. Psychiatr Q.

[22] Verdelho A, Hénon H, Lebert F, Pasquier F, Leys D (2004). Depressive symptoms after stroke and relationship with dementia: a three-year follow-up study. Neurology.

[23] Tatemichi TK, Desmond DW, Stern Y, Paik M, Sano M, Bagiella E (1994). Cognitive impairment after stroke: frequency, patterns, and relationship to functional abilities. J Neurol Neurosurg Psychiatry.

[24] Narushima K, Chan KL, Kosier JT, Robinson RG (2025). Does cognitive recovery after treatment of poststroke depression last? A 2-year follow-up of cognitive function associated with poststroke depression. Am J Psychiatry.

[25] Downhill JE Jr, Robinson RG (1994). Longitudinal assessment of depression and cognitive impairment following stroke. J Nerv Ment Dis.

[26] Ibeneme SC, Nwosu AO, Ibeneme GC, Bakare MO, Fortwengel G, Limaye D (2017). Distribution of symptoms of post-stroke depression in relation to some characteristics of the vulnerable patients in socio-cultural context. Afr Health Sci.

[27] Jobson L, Matharu TK, Kulendran S, Sivakumar VD, Lee QY, Li H, Haque S (2023). Exploring the associations between social support and symptoms of posttraumatic stress disorder among Malaysian and Australian trauma survivors. Eur J Psychotraumatol.

